# Integrated Bioinformatics and Experimental Analysis of Long Noncoding RNA Associated-ceRNA as Prognostic Biomarkers in Advanced Stomach Adenocarcinoma

**DOI:** 10.7150/jca.89526

**Published:** 2024-01-21

**Authors:** Yixin Chen, Xin Gao, Xinyang Dai, Yuwei Xia, Xinran Zhang, Leitao Sun, Ying Zhu

**Affiliations:** 1 The First Affiliated Hospital of Zhejiang Chinese Medical University (Zhejiang Provincial Hospital of Chinese Medicine), Hangzhou 310006, China.; 2 Department of Medical Oncology, The First Affiliated Hospital of Zhejiang Chinese Medical University (Zhejiang Provincial Hospital of Chinese Medicine), Hangzhou 310006, China.; 3 Academy of Chinese Medical Science, Zhejiang Chinese Medical University, Hangzhou 310053, China.; 4 Key Laboratory of Neuropharmacology and Translational Medicine of Zhejiang Province, School of Pharmaceutical Sciences, Zhejiang Chinese Medical University, Hangzhou 310053, China.

**Keywords:** ASTAD, ceRNA network, TCGA, molecular mechanisms

## Abstract

Background: Advanced stomach adenocarcinoma (ASTAD) is a highly malignant and prognostically poor stage of gastric cancer. Recently, long noncoding RNA (lncRNA) was found to play a crucial role, including as competing endogenous RNA (ceRNA) in cancer. However, studies on large-scale sample in ASTAD are still lacking, thus we constructed the ceRNA network of ASTAD to explore its molecular mechanism.

Methods: We compared the expression of mRNAs, lncRNAs and miRNAs between ASTAD and normal tissues utilizing RNA-Seq and miRNA-seq Data from The Cancer Genome Atlas (TCGA). GO and KEGG enrichment analysis were executed for annotating the functions of differentially expressed mRNAs. Subsequently, we investigated the expression correlations between the differentially expressed lncRNAs and their respective mRNAs by constructing a ceRNA network. Kaplan-Meier survival analysis was used to assess the relationship between high/low risk scores based on this network with patient prognosis in TCGA training cohort and GSE15459 validation cohort. In vitro functional assays were employed to verify the cancer-promoting effects of key lncRNAs in the ceRNA network and their possible mechanisms.

Results: In ASTAD tissues, a total of 176 lncRNAs, 124 miRNAs, and 2205 mRNAs were identified as differentially expressed. Our constructed ceRNA network consisted 6 differentially expressed lncRNAs (*PVT1, MAGI2-AS3, KCNQ1OT1, LINC02086, AC125807.2* and *LINC02535*), 25 miRNAs and 130 mRNAs, and the risk score derived from these lincRNAs could predict ASTAD patient outcomes. Key lncRNA LINC02086 was experimentally verified to enhance proliferation and migration of gastric cancer cells by competitively binding to miR-93a-5p with MMP3.

Conclusion: Our comprehensive ceRNA network for ASTAD provides valuable insights into its molecular mechanisms, and LINC02086 may be used as an innovative target for clinical treatment.

## Introduction

In terms of incidence and mortality, gastric cancer is among the top five malignant tumors globally^1^, and it is the third leading cause of cancer-related deaths^2^. Stomach adenocarcinoma (STAD) is the main pathological type of gastric cancer (GC), with poor prognosis and limited treatment options^3^. Chemotherapy remains the primary treatment modality for patients with STAD at stage III or IV, aiming to increase patient survival time and improve quality of life are goals of treatment.

At present, fluorouracil combined with platinum is mostly used as the first-line chemotherapy of ASTAD, but the median Overall Survival (OS) is still difficult to break through 2 years, and the 5-year survival rate is less than 20%^4^. Molecular targeted therapy is an emerging treatment in recent years. The ToGA trial showed that the addition of trastuzumab to chemotherapy can increase the median OS to 16 months and the response rate to 47% in HER-2 positive patients^5^. However, the positive rate of HER-2 in STAD patients is only about 22%^6^, limiting the potential benefit of trastuzumab to a small subset of patients. Moreover, STAD exhibits high heterogeneity, leading to unsuccessful outcomes in several other targeted drug therapy studies^7^. Although in recent years, immunotherapy has been as another treatment strategy for cancer, finding from the Keynote-062 study revealed that combining anti PD-L1 with chemotherapy didn't provide additional efficiency^8^. So, immunotherapy remains a challenge in the first-line treatment of ASTAD. In order to comprehend the molecular process of ASTAD and look for novel molecular targets, it is crucial to explore ASTAD biomarkers and use risk stratification and prognostic prediction using biomarkers to develop new and more effective treatment regimens.

Non-coding RNA is an increasing important direction of current molecular research, including types of miRNA, lncRNA and circRNA. For lncRNAs (longer than 200 nucleotides to 100 kb in length with little or no protein-coding potential), it can work as ceRNA, also known as RNA sponge by binding to miRNAs. In malignant tumor cells, ceRNAs can weaken miRNA inhibition on targeted mRNAs, playing a significant role in tumor occurrence, progression, metastasis, and even drug therapy^9^.Differential expression of lncRNAs in tumors can help identify their competing mRNAs and enhance our understanding of their significance. For example, studies have shown that lncRNA HOXC-AS1 targets miR-99a-3p and upregulates MMP8 to promote gastric cancer^10^, lncRNA ELFN1-AS1 upregulates TRIM29 by inhibiting miR-211-3p to accelerate the growth of GC,^11^, and the interaction between lncRNA MIR100HG and hnRNPA2B1 contributes to colorectal cancer advancement. 12.

In tumors, different lncRNAs may compete for the same miRNA, and lncRNAs also share a certain number of miRNAs with mRNAs, thereby forming complex RNA regulatory networks that affect pathways and functions. While some studies have constructed lncRNA-associated ceRNA networks in GC,^13^ it is urgent to build a ceRNA network specific to ASTAD, select key lincRNA, verify their molecular biological functions and ceRNA mechanism of action, and apply them in treatment decision-making, prognosis prediction and targeted therapy of advanced gastric adenocarcinoma.

## Materials & Methods

### Data collection

The clinical data on patient age, sex, and race were extracted from the STAD dataset in the TCGA database, from which we selected patients with TNM stage III or IV for further data analysis. The inclusion criteria were that: (i) the histological diagnosis was STAD; (ii) sufficient information for clinical characteristics (including age, gender, race, stage, survival status and survival time); and (iii) the AJCC pathologic stage were III or IV. In total, 186 ASTAD patients were included in this study and their clinical characteristics were summarized in Supplemental Table 1.

### Explore the differentially expressed genes

The RNA sequencing (RNA‐Seq) and miRNA sequencing (miRNA‐Seq) data were derived from the TCGA data portal. In total, we analyzed data from 177 ASTAD tumor tissues and 9 adjacent normal tissues were available for RNA-seq, including lncRNA-Seq and mRNA-Seq. Additionally, miRNA-Seq information was available for 178 ASTAD tumor tissues and 7 adjacent normal tissues. To identify significantly differentially expressed mRNA (DEG), lncRNA (DELnc) and miRNA (DEM) between cancer tissues and normal tissues, we utilized the edgeR package in R software^14^. The cut‐off value was |log_2_FC| > 1 and FDR <* 0.*05 (FC, fold change; FDR, adjust p value).

### Function and pathway enrichment

The gene ontology (GO) and Kyoto Encyclopedia of Genes and Genomes (KEGG) analysis were necessary to better understand the function of differentially expressed genes. GO enrichment analysis^15^ was mainly used to annotate the genes and gene products, with terms classified into three categories: biological processes (BP), cellular composition (CC), and molecular function (MF). The KEGG^16^ database contains information on genomes, biological pathways, diseases, and chemical compounds. Therefore, for differentially expressed RNA (DEG and DELnc), we used enrichGO and enrichKEGG packages in R for functional analysis. Next, the pathview packages in R was used to construct KEGG pathways.

### Construct the competitive endogenous RNA network

The gdcCEAnalysis package in R was used to predict the interactions between lncRNA-miRNA and mRNA-miRNA. Statistically significant correlations were determined by performing correlation analysis with p < 0.05 and regsim correlation coefficient not equal to zero. We employed the Starbase database^17^ online for verification and supplementation of miRNAs predicted to be targeted by the DElncs. And the mRNA-miRNA pairs were supplemented with miRTarBase^18^ and TargetScan^19^ databases. The resulting interactions were then utilized to construct the lncRNA‐miRNA‐mRNA network using Cytoscape (version 3.7.0)^20^.

### Correlation analysis and survival analysis

The correlation between lncRNAs and their corresponding mRNAs was analyzed using the gdcCorPlot package in R. To assess the prognostic relevance of competitive endogenous RNA, we integrated the clinical data from ASTAD patients. Based on the median expression value of each lncRNA in ASTAD patients, we used the gdcKMPlot package in R to draw KM survival curves to show the correlation between the expression level of each lncRNA in the ceRNA network and patients' prognosis.

### CeRNA Network in relation to overall survival of the ASTAD patients

For the DElncRNAs in the ceRNA network, we conducted multivariate COX regression in SPSS 25.0. Subsequently, we calculated the prognostic risk score as: exp_lncRNA1_ * β_lncRNA1_+ exp_lncRNA2_ * β_lncRNA2_ + exp_lncRNAn_ * β_lncRNAn_ (exp, expression level; β, the regression coefficient derived from the multivariate Cox regression model) 21. The TCGA-ASTAD data was used as the training cohort. Additionally, we selected the GSE15459 dataset from GEO database and screened data of patients at stages 3-4 to serve as an external validation cohort. The risk scores of the two cohorts were calculated, then the high-risk group and the low-risk group were respectively divided accordingly in each cohort. The overall survival rates of the high-risk and low-risk groups ware compared by KM analysis using "ggsurvplot" package. Furthermore, time-dependent ROC curves were generated using the "survivalROC" package with a focus on predicting 1-, 2-, and 3-year survival in ASTAD patients based on the "NNE" method^22^.

### Cell culture

The human gastric cancer cell lines MKN-1, HGC-27, NUGC-3, NUGC-4, human normal gastric epithelial cell GES-1 and human embryonic kidney (HEK) 293T cell were obtained from Key Laboratory of Prevention, Diagnosis and Therapy of Upper Gastrointestinal Cancer of Zhejiang Province. The STR fingerprint authentications can be provided upon request. The above cell lines were cultured in Roswell Park Memorial Institute (RPMI) 1640 (BI, Israel) or Dulbecco's modiffed Eagle's medium (DMEM; Cienry, China) medium supplemented with 10% fetal bovine serum (FBS, MEILUNCELL, China), and 1% penicillin and streptomycin in a 5% CO2 incubator (ThermoFisher, USA) at 37℃ humidified environment.

### RNA interference

Small interfering RNAs (siRNA) directed against LINC02086 and negative control (siNC) were synthesized by Zhejiang Sunya Company (Hangzhou, China). Transient transfection was performed by Polyplus jetPRIME kit (France) on NUGC-3 cells following standard protocol. We harvested cells after 48 h for RT-qPCR examination and functional assays or after 72 h for Western Blot analysis. The siRNA sequences were generalized as flowing: si-LINC02086#1 sense: CCUUGGAGGUCUGAUGCAUUUTT; si-LINC02086#1 antisense: AAAUGCAUCAGACCUCCAAGGTT; si-LINC02086#2 sense: AAGGCUGGUCCUGUCUCUAUATT; si-LINC02086#2 antisense: UAUAGAGACAGGACCAGCCUUTT.

### Cell proliferation assay and colony formation

Cell proliferation ability was assayed by Cell Counting Kit-8 (CCK-8, MedChemExpress, USA). NUGC-3(5× 10^3^ cells/well) were seeded in 96-well plates and incubated at 37 °C for one hour. Absorbance at 450 nm was measured daily for 4 consecutive days. For the colony formation assay, treated NUGC-3 cells (5× 10^3^ cells) of each group were inoculated in 6-cm dishes and incubated for at least one week. After washing with phosphate-buffered saline (PBS), the cells were fixed in methanol for 30 min and stained with 0.1% crystal violet solution for 30 min before analysis.

### Cell cycle assay

The transfected NUGC-3 cells in each group were collected and fixed in 75% ethanol at - 20°C overnight. On the next day, after discarding the ethanol, the cells were washed once with PBS, resuspended with 500 μl DNA staining solution (Multiscience, China), and stained for 30 min at room temperature. Cell cycle assay was performed using flow cytometry (FACS LSRII, BD Bioscience, USA) and the results were analyzed using ModfitLT 5.

### Edu assay

We used the 5-ethynyl-20-deoxyuridine (EdU) assay kit (Beyotime, Shanghai, China) to de-duplicate the cell proliferation ability. 1.2 × 10^5^ NUGC-3 cells from each group were inoculated into confocal plates and incubated with 10 μM EdU buffer at 37 °C for 2 h, then fixed with 4% formaldehyde for 30 min and permeabilized with 0.1% Triton X-100 for 30 min. The nuclei of the cells were stained with Hoechst staining after the addition of Click additive solution to the culture medium. Finally, the results were observed by fluorescence microscope.

### Cell migration ability assay

Transwell assay was conducted using 24-well plates with 8-μm pore size transwell filter inserts (Corning, USA). A total of 1.2 × 10^5^ NUGC-3 cells were suspended using serum-free medium and added to the upper chamber. Medium containing 10% FBS was added to the lower chamber. After incubation in 37 °C for 24h, the cells on the underside of membrane were fixed and stained with Wright's-Giemsa stain (Nanjing Jiancheng, China) and further photographed.

For the wound healing experiment, the 2-well Culture-Insert (Ibidi, Germany) was pre-inserted in a 12-well plate. Then, treated cells were inoculated into each compartment at a density of 0.7 million cells and incubated at 37°C for 24h. The culture inserts were removed for scratch formation, and then the cells were photographed and continued to be cultured in ≤ 1% FBS medium to allow them to migrate and gradually close the wounds, and photographed under the microscope every 24h.

### Total RNA extraction and RT-qPCR

Total RNA was isolated from GC cell lines using FastPure Cell/Tissue Total RNA Isolation Kit (Vazyme Biotech, Nanjing, China). Subsequently, 1ug of RNA underwent reverse transcription into cDNA using the HiScript II Q RT SuperMix (Vazyme Biotech, Nanjing, China). The fluorescence-based SYBR Green (Vazyme Biotech, Nanjing, China), detected on the ABI QuantStudio 5 Real-Time system (Applied Biosystems, USA). The relative RNA amount was calculated using the 2^-ΔΔCt^ method, normalized to β-actin.

All premiers were derived from Tsingke Biological Technology (Beijing, China) and were displayed as following: β-actin, F: 5'-CATCCACGAAACTACCTTCAACTCC-3', R: 5'-GAGCCGCCGATCCACACG-3'; LINC02086, F: 5'-CTGTTACAGCAGAACCGAAGATTT-3', R: 5'-GGCAAATGAAATCCCAGACAG-3'; LINC02535, F: 5'-CAGGCTTTGTATCTTCAGTTGCTT-3', R: 5'-GGTTGAGGTGGAATCGAGTGTT-3'.

### Western blotting

Total protein was extracted using RIPA buffer (Fdbio science, Hangzhou, China) containing 1% protease and phosphatase inhibitors (Bimake, USA). Protein quantification was performed using BCA Protein Assay Kit (Fdbio science, Hangzhou, China). Protein samples of 40ug from each group were separated by 4-20% SDS-PAGE (GenScript, Nanjing, China) and then transferred to 0.22 μm PVDF membranes (Millipore, USA). Following a 2-hour blocking step in 3% Bovine Serum Albumin (BSA) at room temperature, the membranes were incubated overnight at 4℃ with primary antibodies including anti‐β-actin (1:5000; YM3028, Immunoway), anti-MMP3 (1:1000, ab52915, Abcam), anti‐E‐cadherin (1:500; YT1454, Immunoway), anti‐ZO-1 (1:500; YN1410, Immunoway), anti-N-cadherin (1:500, YT2988, Immunoway), anti-β-Catenin (1:1000, YM3403, Immunoway), anti-Snail (1:500, YT4351, Immunoway) and anti-Vimentin (1:500, YT4880, Immunoway). The next day, the membranes was washed 3 times with TBST and incubated with HRP-secondary antibody for 2 hours at room temperature. Immunoblots were detected with an imaging system (Bio-Rad, USA) and an enhanced chemiluminescence detection kit (Fdbio science, Hangzhou, China). GAPDH was selected as a loading control.

### Dual luciferase reporter assay

The wild-type (WT) or mutant-type (MUT) sequences of LINC02086 and 3' UTR of MMP3 were cloned into the pmirGLO plasmid (REPOBIO, Hangzhou, China). The miR-93-5p mimics and corresponding negative control were synthesized by RiboBio (Guangzhou, China). The interaction between miR-93-5p and its putative binding sites was tested by the Dual Luciferase Reporter Assay Kit (Vazyme Biotech, Nanjing, China). Briefly, HEK293T cells or NUGC-3 cells were seeded in a 24-well plate and co-transfected with 300 ng of the luciferase reporter constructs (pmirGLO plasmid) and miR-93-5p mimics (20uM), or with si-LINC02086 (20uM) or overexpressed LINC02086 plasmid (300ng) using Polyplus jetPRIME kit. After 48 h, cells were lysed, incubated with firefly and Renilla luciferase substrates, and measured for enzyme activity using a luminometer. Firefly luciferase activity was normalized against Renilla luciferase to represent miRNA binding efficiency. Each group was conducted in triplicate.

### Statistical analysis

Each experiment was repeated independently 2-3 times and the results were analyzed using GraphPad Prism 9.0. Measurement data were expressed as mean ± SD. One-way analysis of variance (ANOVA) or t-test was used to compare quantitative data, and nonparametric χ2 test was used to compare qualitative data.* P*-values for each analysis are marked on figures, and the level of statistical significance was defined to *P* < 0.05 (*P < 0.05; **P < 0.01; ***P < 0.001; ****P < 0.0001).

**Figure 1:**
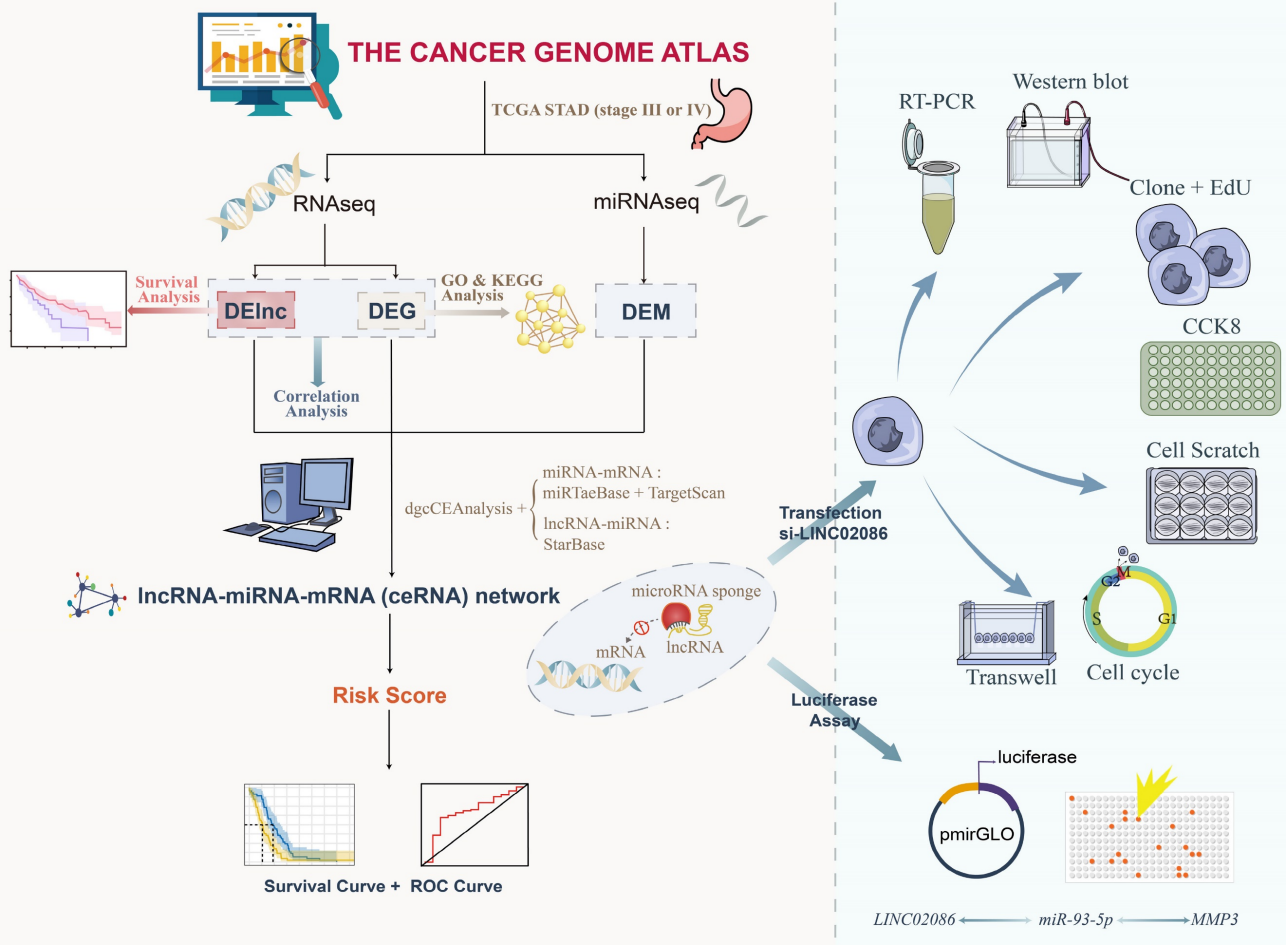
Flowchart for study.

Above, research flow was shown in Figure 1.

## Results

### Clinical characteristics of advanced stomach adenocarcinoma patients

In the analyzed dataset, 186 ASTAD patients were enrolled in the study, with 148 (79.57%) at stage III and 38 (20.43%) at stage IV. Among them, 125 (67.20%) were aged ≤70 years while the remaining 61(32.80%) were over 70 years old, with a median age of 65.23 years old. Of these patients, there were more males (62.37%) than females (37.63%). The ethnic distribution was as follows: Asian-30 (16.13%), Black or African American-7 (3.76%), White-118 (63.44%), Native Hawaiian or other Pacific Islanders-1 (0.54%), and not reported-130 (16.13%). Only six patients had a duration of illness ≥ 5 years. The detail results were listed in Supplemental Table 1.

### Differentially expressed lncRNA, miRNA and mRNA

**Figure 2:**
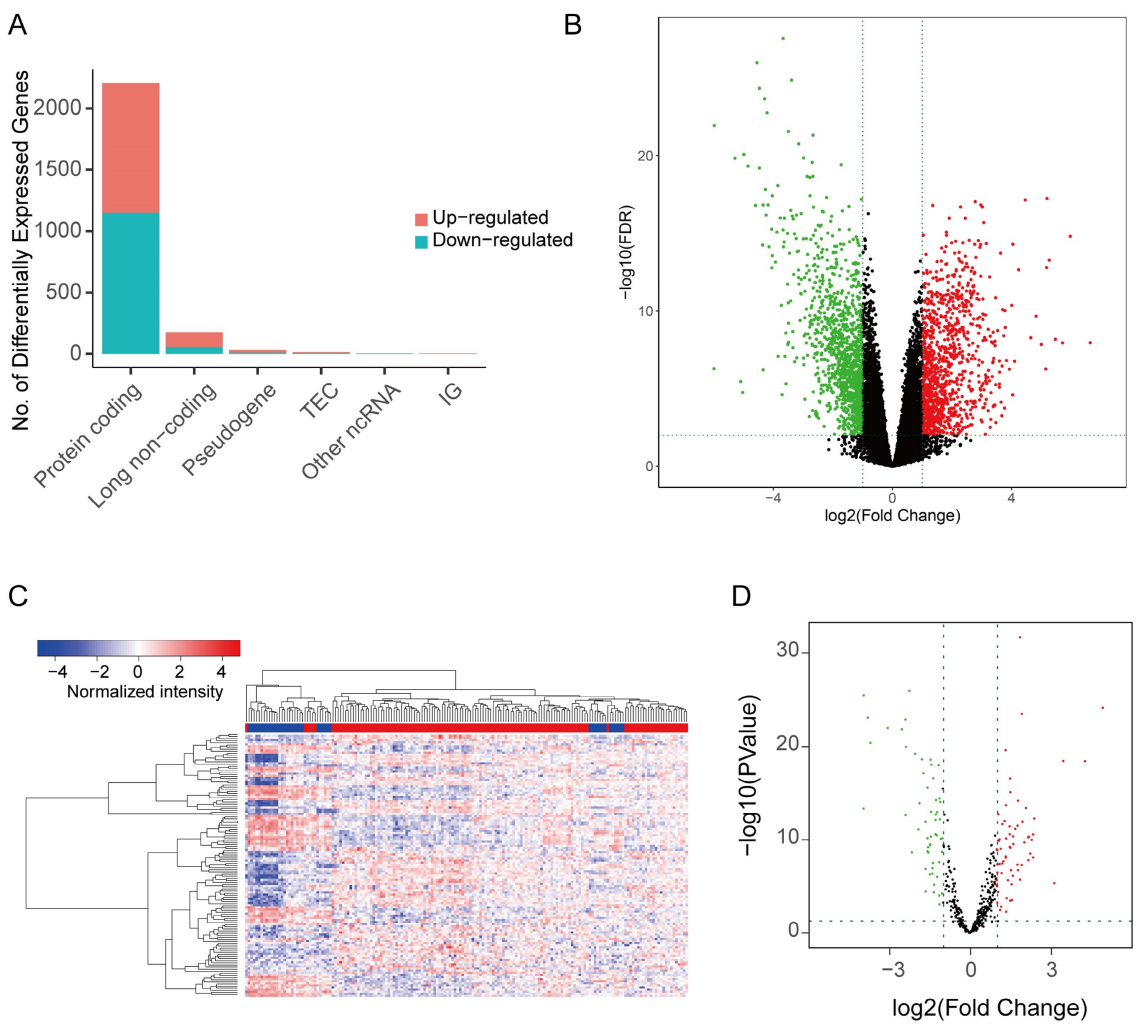
The DEG and DELnc between ASTAD tumor tissues and normal tissues was explored from TCGA database. (A-B) The analysis identified 2205 protein coding RNAs and 176 lncRNA (C-D) Filtering analysis identified 124 DEM between normal tissues and cancer tissues.

The study investigated differentially expressed genes (DEG) and differentially expressed lncRNA (DELnc) in tumor tissues compared to normal tissues. |log2FC| > 1 and FDR <* 0*.05 were considered as discriminatively expressed. The analysis identified 2205 protein coding RNAs (1056 elevated and 1149 downregulated) and 176 lncRNA (121 elevated and 55 downregulated) (Figure 2A-B). Similarly, filtering analysis based on the above criteria identified 124 differential expression miRNAs (DEM), including 68 up-regulated and 56 down-regulated, between cancerous and normal tissues (Figure 2C-D). These findings suggested that the expression patterns of these genes can serve as distinguishing factors for ASTAD from normal tissues.

### GO and KEGG enrichment analysis of DEG

**Figure 3:**
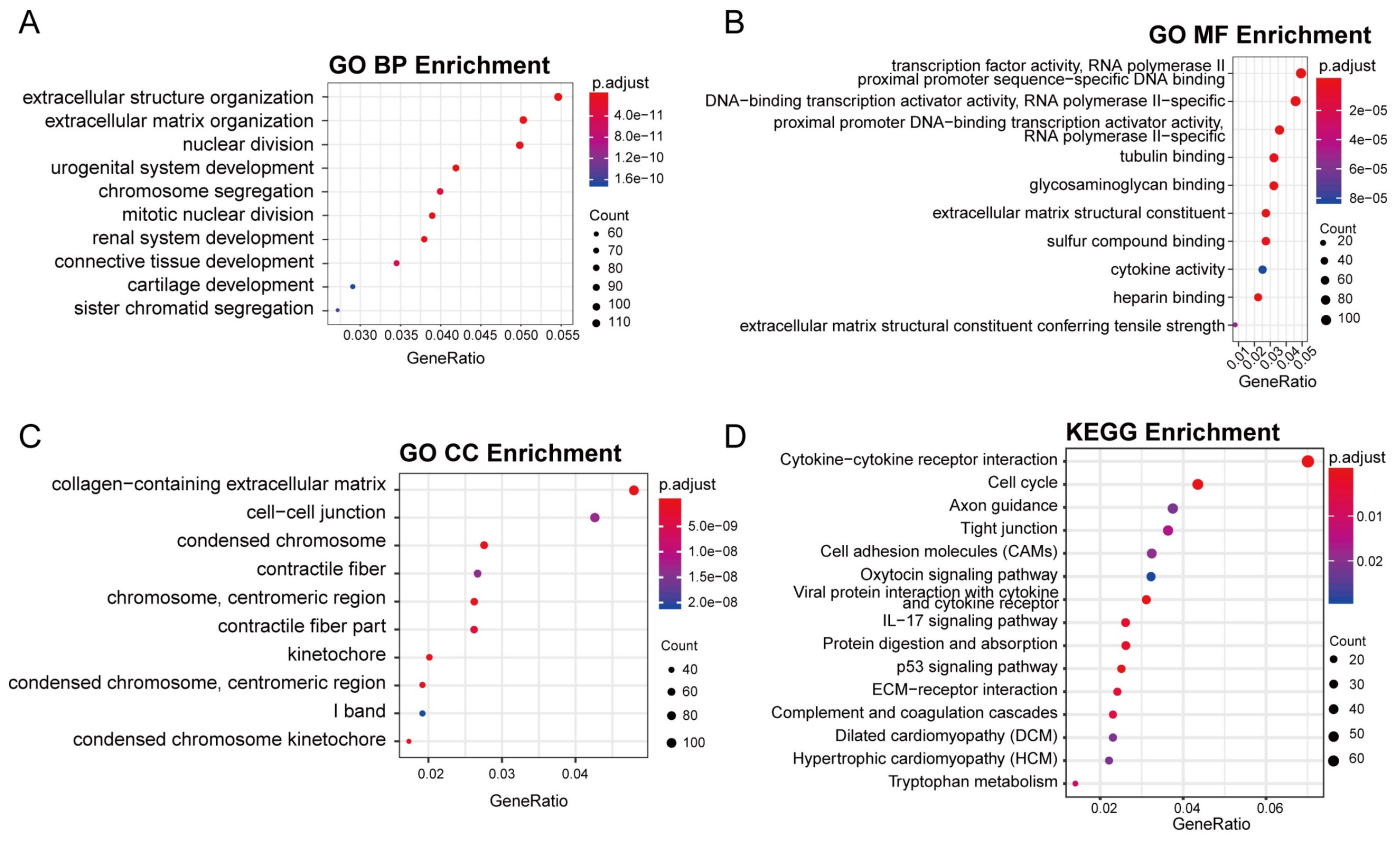
The result of GO and KEGG enrichment analysis of DEGs. (A) The first 10 items of biological process category sorted by p-value in the GO analysis. (B) The first 10 items of molecular function category sorted by p-value in the GO analysis. (C) The first 10 items of cellular component category sorted by p-value in the GO analysis. (D) The first 10 items of KEGG enrichment sorted by p-value.

The GO analysis revealed that most of the differentially expressed genes (DEGs) were involved in various biological processes, including extracellular matrix and structure organization, cell proliferation (sister chromatid segregation and nuclear division), and tissue development (cartilage, issue, renal system, etc.) (Figure 3A). Most of these biological processes occur in regions such as the extracellular matrix and chromosomes (Figure 3C). Accordingly, the molecular functions of DEGs mainly include in gene expression process such as transcription factor activity, cell proliferation process and RNA polymerase II proximal promoter sequence-specific DNA binding (Figure 3B). In KEGG enrichment analysis, the DEGs were predominantly associated with pathways like Cell cycle, Cytokine-Cytokine receptor interaction, Viral protein interaction with Cytokine and Cytokine receptor and other signaling pathways (Figure 3D), consistent with the findings from GO analysis. Notably, the cell cycle pathway had the most significant p-value (Supplemental Figure 1).

### Competitive endogenous RNA network in Advanced stomach adenocarcinoma

The potential interactions among the DEG, DELnc and DEM were predicted based on the ceRNA hypothesis. Using the gdcCEAnalysis package in R and the supplementary prediction through the Starbase database, miRTarBase and TargetScan databases online, we generated a ceRNA network model consisting of 6 DELncs, 25 DEMs and 130 DEGs interacting with each other. Table 1 presents the mutual prediction results between LncRNA-miRNA and miRNA-mRNA.

**Figure 4:**
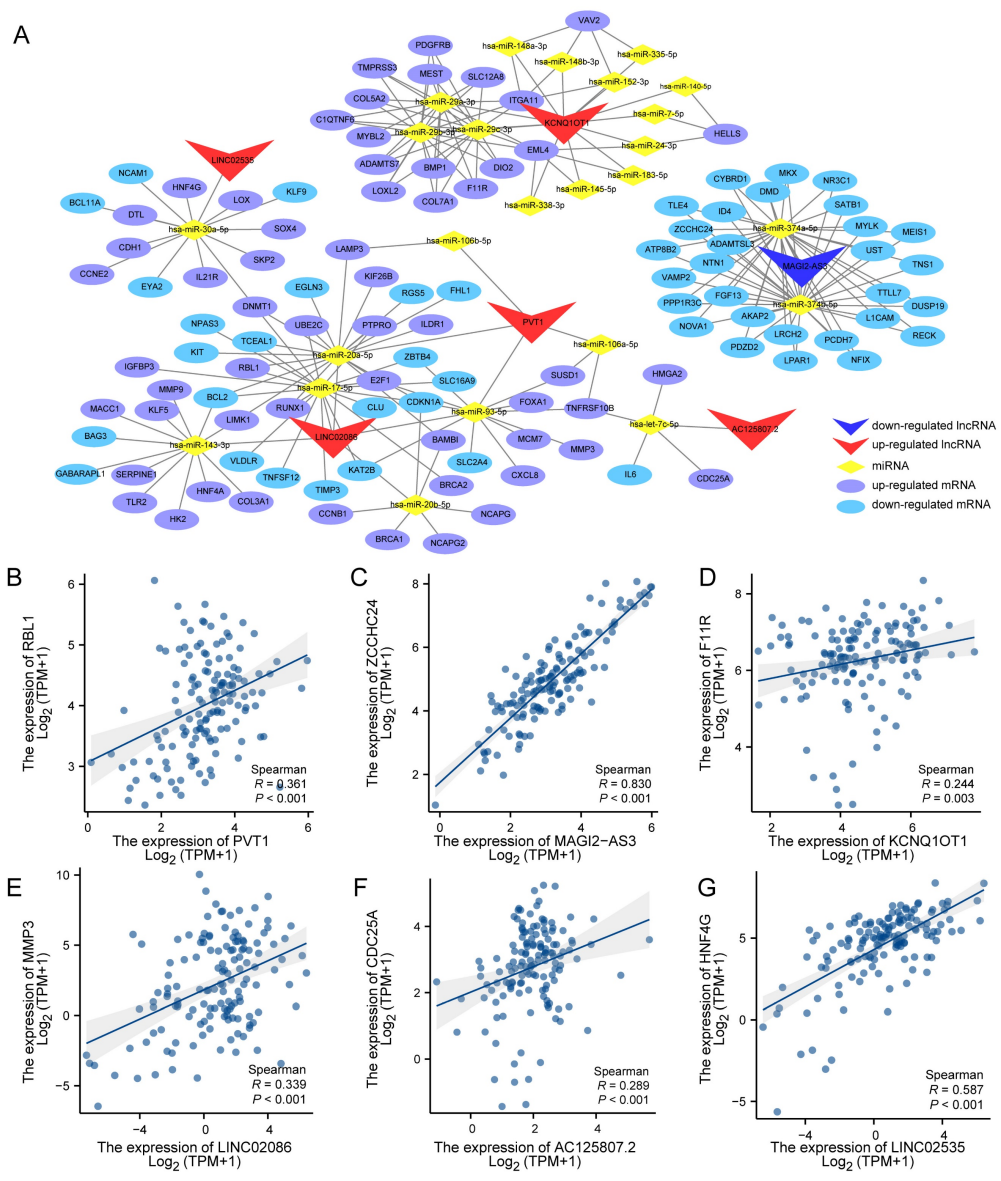
Competitive endogenous RNA network in Advanced stomach adenocarcinoma and correction analysis between DElnc and DEM. (A) The lncRNA-miRNA-mRNA (ceRNA) network model of ASTAD constructed containing 6 DELnc, 25 DEM and 130 DEG interacting with each other. (B-G) The correlation analysis between each LncRNA in the ceRNA network and its mRNA with the highest expression correlation coefficient.

Next, the interactions of these results were used to construct the ceRNA network utilizing the Cytoscape_v3.7.0 (Figure 4A). According to the ceRNA action model, both lncRNA and mRNA competitively bind to the same miRNA, leading to mRNA inhibition. Consequently, a positive expression correlation is expected between lncRNA and mRNA. Therefore, we performed expression correlation analysis for six lncRNAs and their respective competitive mRNAs within the aforementioned ceRNA network. The results displayed scatter plots illustrating each lncRNA's highest expression correlation coefficient with its corresponding mRNA (Figure 4B-G).

### Survival analysis

**Figure 5:**
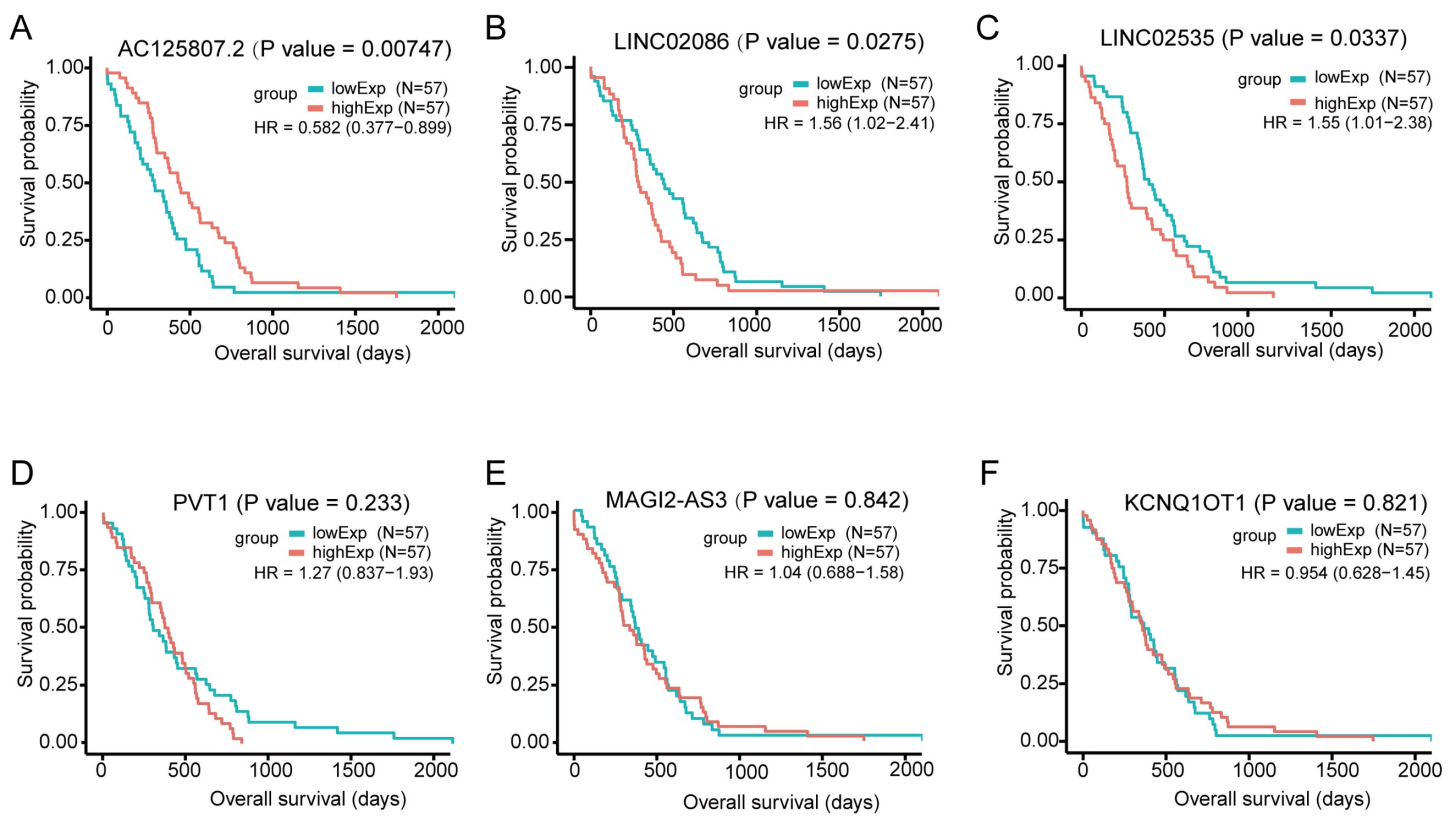
Overall survival analysis for the differentially expressed lncRNAs in the ceRNA network in patients with ASTAD, based on the median expression value of each lncRNA. It showed *AC125807.2, LINC02086* and *LINC02535* had statistically significant survival curves. (A) KM survival curve for *AC125807.2.* (B) KM survival curve for *LINC02086.* (C) KM survival curve for *LINC02535.* (D) KM survival curve for *PVT1.* (E) KM survival curve for *MAGI2-AS3.* (F) KM survival curve for *KCNQ1OT1.*

For the DElncs in the ceRNA network, we used the gdcKMPlot package in R for univariate KM analysis to estimate the effect of lncRNAs on overall survival in patients with ASTAD. Only 114 individuals with available survival time information were included in the analysis. The results of KM analysis revealed that out of these 6 lncRNAs, only three exhibited statistically significant survival curves: *AC125807.2, LINC02086* and *LINC02535* (Figure 5). The expression of *AC125807.2* was positively correlated with OS of ASTAD patients, while *LINC02086* and *LINC02035* were negatively.

**Figure 6:**
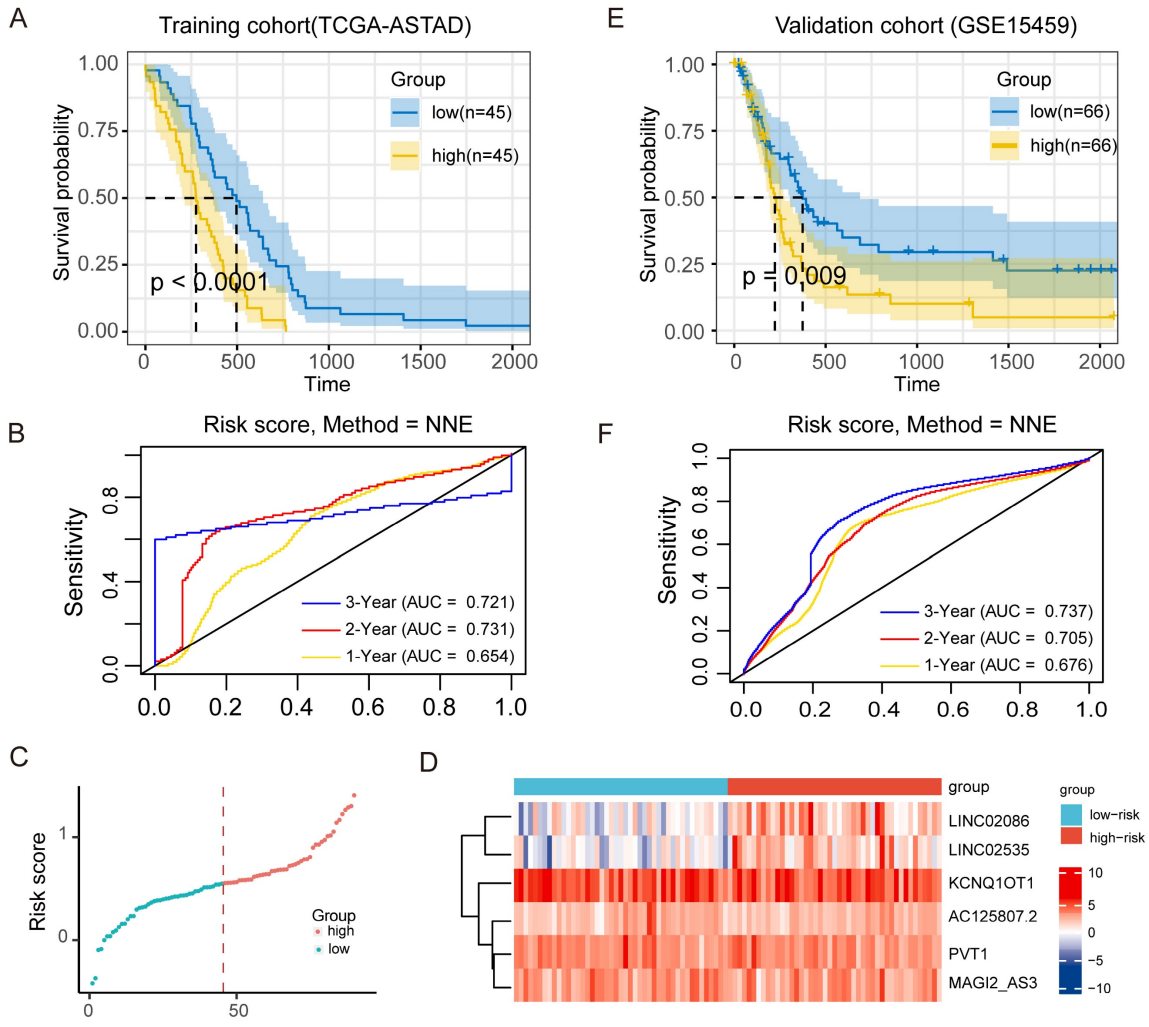
Prognostic effect of ceRNA network model on ASTAD patients. (A) KM survival curve for patients with high-risk scores and low-risk scores in training cohort. (B) ROC curve for assessing the efficacy of our signature in predicting patients' 1-year, 2-year, and 3-year survival rates in the training cohort. (C) The scatter diagram of the risk score and the expression of 6 lncRNAs in the entire cohort. (D) The scatter diagram of the risk score and the heatmap of risk score and the expression of 6 lncRNAs in the entire cohort. (E) KM survival curve for patients with high-risk scores and low-risk scores in validation cohort. (F) ROC curve for assessing the efficacy of our signature in predicting patients' 1-year, 2-year, and 3-year survival rates in thevalidation cohort.

Further, for the TCGA-ASTAD training cohort, according to COX regression coefficient, the risk score of lncRNA-ceRNA network was calculated as follows: the expression of LINC02086 * 0.056 + the expression of LINC02535 * 0.082 + the expression of AC125807.2 * (-0.231) + the expression of PVT1 *0.0181 + the expression of MAGI2-AS3 * 0.109 + the expression of KCNQ1OT1 * (-0.007), and the median risk score in it was 0.561. Similarly, the risk score of the GSE15459 validation cohort was: LINC02086 * 0.025 + LINC02535 * 0.042 + AC125807.2 *0.244+ PVT1 * 0.232 + MAGI2-AS3 * 0.116 + KCNQ1OT1 *0.018, and the median risk score was 5.497. Then, patients in two cohorts were categorized into low-risk and high-risk groups based on median risk score. KM survival curves were subsequently employed to assess the OS of the different risk groups. The result showed significantly poorer survival in the high-risk patients in the training cohorts (*P* <0.0001; Figure 6A), and the result of the time-dependent ROC curve show that the signature has a good predictive efficacy on the patients' 1-year, 2-year 3-year survival rates (Figure 6B, the area under the curve (AUC) were 0.654, 0.731 and 0.721, respectively). Meanwhile, the scatter diagram of the risk score and the heatmap of risk score and the expression of 6 lncRNAs in the entire cohort are shown in Figure 6C and Figure 6D. The results of the heatmap showed that the expression of *LINC02086* and *LINC02535* was basically in line with high expression in high-risk tissues and low expression in low-risk tissues, while there was no significant difference in the distribution of the other four lncRNAs. In addition, the KM curve of the external validation cohort (GSE15459) also confirmed that high-risk patients had significantly worse prognosis compared to their low-risk counterparts (*P* = 0.009; Figure 6E). As shown in Figure 6F, the risk signature can also accurately predict the survival rate in the validation cohort, whose 1-year, 2-year and 3-year area under the ROC curve are 0.676, 0.705, and 0.737, respectively.

### LINC02086 acting as a ceRNA, competitively binding miR-93-5p with MMP3, and promoting GC progression in vitro

**Figure 7:**
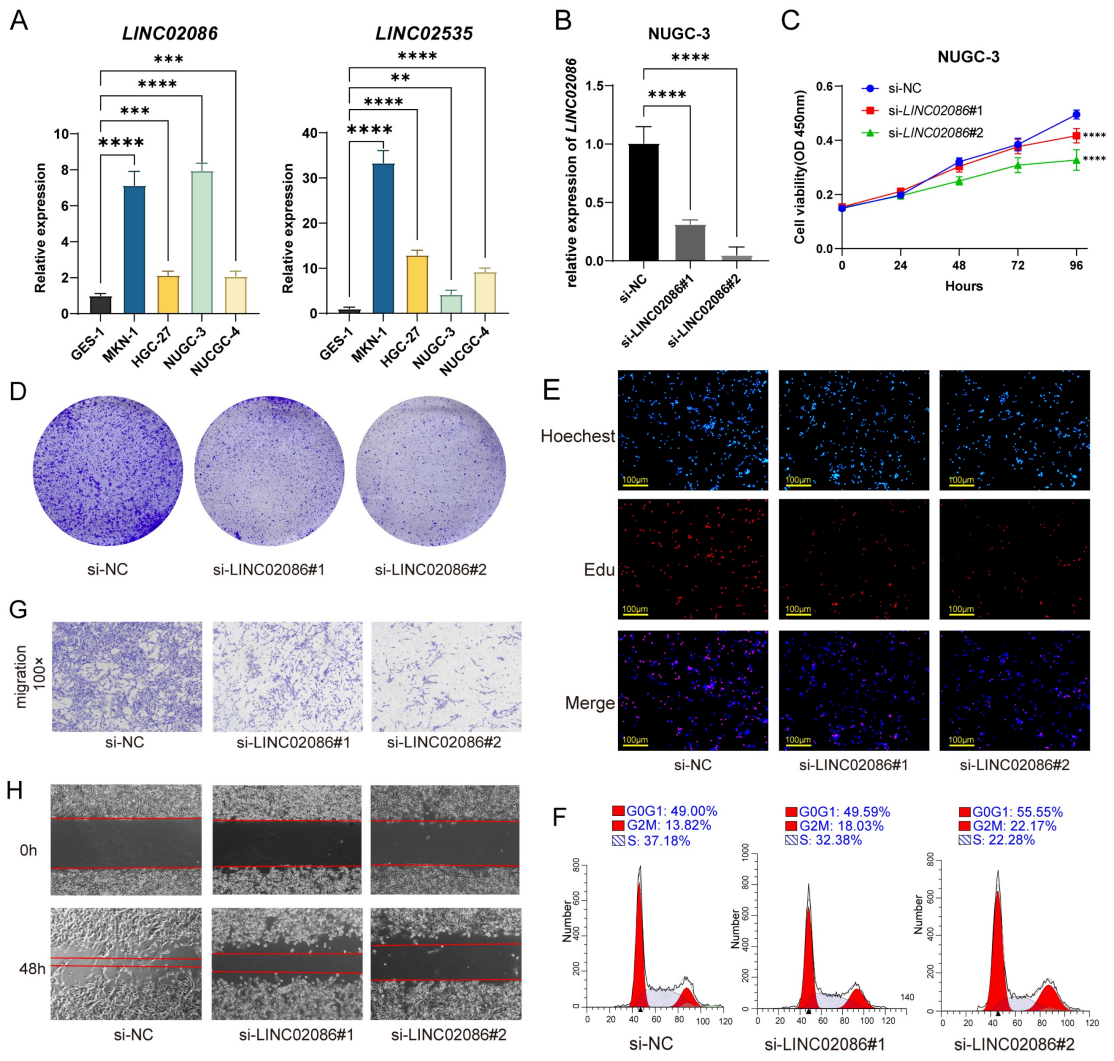
The verification of LINC02086 promoting GC progression in vitro. (A) RT-qPCR showed the expression levels of* LINC02086* and *LINC02535* were significantly higher in gastric cancer cells then normal gastric epithelial cell. (B) RT-qPCR showed the knockdown efficiency of siLINC02086 in NUGC-3 cell. (C-E) CCK-8, clonal formation assay and EdU assay showed that the decreased expression of LINC02086 inhibited the proliferation ability of gastric cancer cells. (F) Cell cycle detection results showed that the down-regulated expression of LINC02086 caused certain G2 phase arrest of gastric cancer cells. (G-H) The transwell and scratch assays showed that the expression of LINC02086 was significantly correlated with the migration ability of gastric cancer cells.

On one hand, due to the prognostic correlation of LINC02086 and LINC02535 in ASTAD, RT-qPCR was used to detect their expression levels between GC cells and normal gastric epithelial cell. The results revealed a significant upregulation of LINC02086 and LINC02535 in GC cells (Figure 7A). After siRNA transfection was used to knock down the expression of LINC02086 in GC cell NUGC-3, it showed the changes in the cell function. Specifically, CCK-8, clonal formation and EdU assay showed that the decreased expression of LINC02086 inhibited the proliferation ability of GC cells in vitro (Figure 7C-E), while cell cycle detection indicated a certain G2 phase arrest (Figure 7F). Furthermore, transwell and scratch assays confirmed that the expression of LINC02086 was significantly correlated with the migration ability of gastric cancer cells (Figure 7G-H).

**Figure 8:**
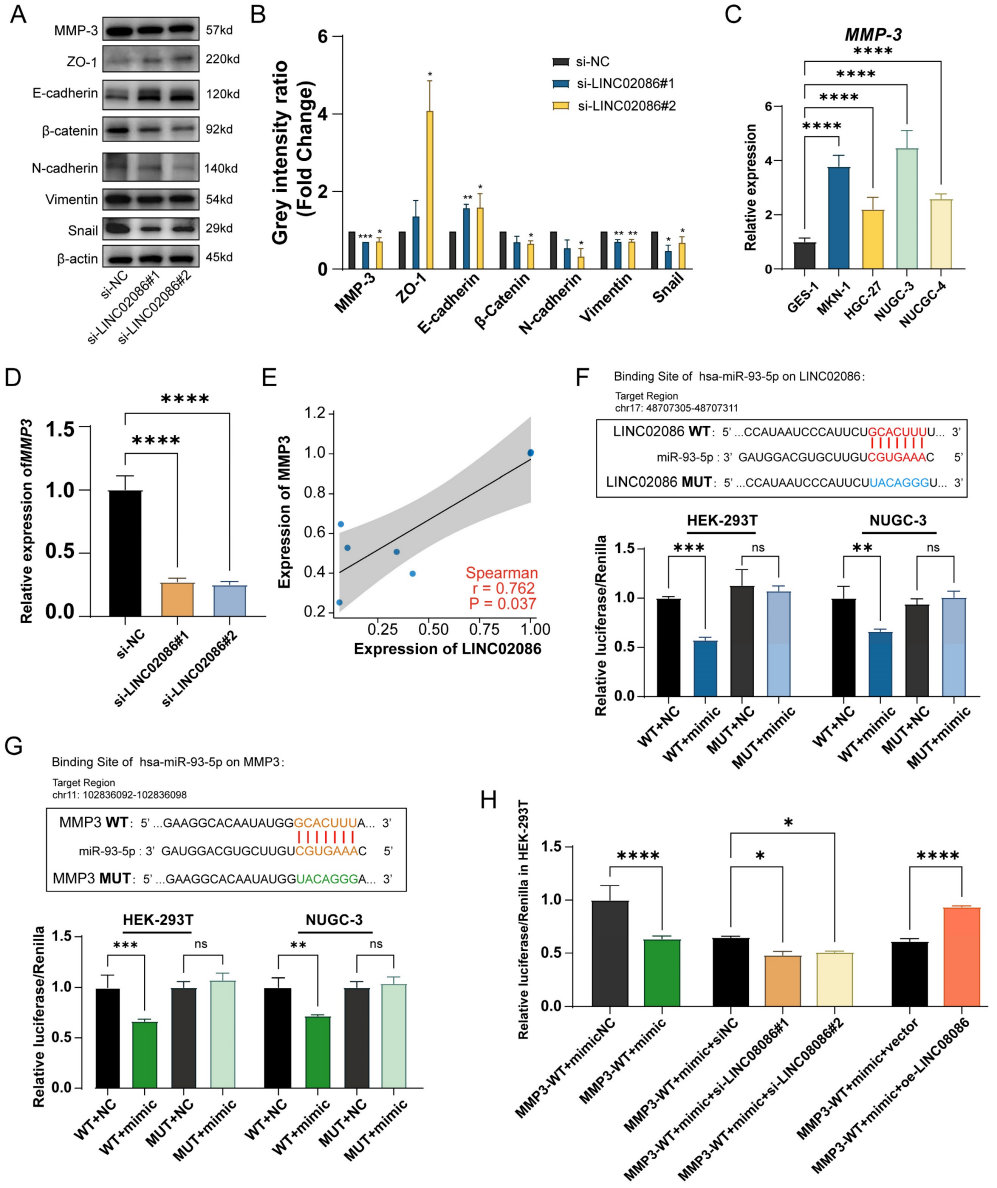
The verification of LINC02086 acting as a ceRNA, competitively binding miR-93-5p with MMP3. (A) Western blot showed the EMT-related markers and *MMP3* were associated with LINC02086 expression. (B) The grey intensity ratio of western blot results. (C) RT-qPCR showed the expression levels of* MMP3* were significantly higher in gastric cancer cells then normal gastric epithelial cell. (D) RT-qPCR showed that the mRNA level of *MMP3* was down-regulated in si-LINC02086 group. (E) Scatter plot of expression correlation between *MMP3* and *LINC02086* at mRNA level based on PCR results. (F) Dual-luciferase reporting assay verified the target binding of LINC02086 and miR-93-5p. (G) Dual-luciferase reporting assay verified the target binding of MMP3 and miR-93-5p. (H) Dual-luciferase reporting assay showed the Luciferase activity of miR-93-5p on MMP3 influenced by the si/oe-LINC02086.

In addition, western blot showed that EMT-related markers also had corresponding expression changes in the si-LINC02086 group. Specifically, the expression of epithelial markers such as E-cadherin and ZO-1 was up-regulated, while that of interstitial markers such as N-cadherin, β-catenin, Snail and Vimentin were down-regulated (Figure 8A-B). These findings comprehensively prove that high LINC02086 expression in gastric cancer promotes cell growth, proliferation and migration in vitro.

On the other hand, we analyzed mRNAs in the ceRNA network and identified MMP3 as the most significantly differentially expressed gene in ASTAD patients from the TCGA dataset (Supplemental Table 2). In the ceRNA network, both MMP3 and LINC02086 were likely to bind to miR-93-5p. Therefore, we propose that LINC02086/miR-93-5p/MMP3 axis might play a role in promoting ASTAD through the regulatory mechanism of ceRNA. In order to verify the above possibility, we performed RT-qPCR and WB detection on si-LINC02086 GC cell, and the results demonstrated that inhibition of LINC02086 expression led to down-regulation of both RNA (Figure 8C) and protein levels (Figure 8A-B) of MMP3. This confirmed a correlation between LINC02086 expression and MMP3 (Figure 8E). Furthermore, dual luciferase reporting assays confirmed direct regulatory effects of miR-93-5p on both LINC02086 and MMP3 through binding at the same site (Figure 8F-G). In addition, after the combination of si-LINC02086, the regulatory effect of miR-93-5p on MMP3 was weakened, and on the contrary, it was enhanced in the oe-LINC02086 group (Figure 8H). These findings provide further evidence for the involvement of LINC02086 as a ceRNA in gastric cancer.

## Discussion

There have been many studies of the ceRNA network in various cancers, including stomach cancer. However, few of them have focused on the different stages with different prognosis, whose biomarkers should also be different. ASTAD has its high heterogeneity and lacks mature targeted therapy and immunotherapy, thus requiring more relevant biomarkers to find new therapeutic targets and play their clinical role. Large-scale sample studies focusing specifically on ASTAD using microarray detection are still lacking. Hence, we selected patients with pathological stage III-IV from TCGA-STAD patients to analyzed the expression patterns of ASTAD mRNA, miRNA and lncRNA using microarray data. Despite limited sample availability (9 RNAseq and 7 miRNASeq data from adjacent normal tissues), we included all available sequencing data in our analysis to maximize sample utilization. Based on differentially expressed mRNA-miRNA and miRNA-lncRNA pairs, we constructed a ceRNA network comprising *PVT1, MAGI2-AS3, KCNQ1OT1, LINC02086, AC125807.2* and* LINC02535* as the ceRNAs.

Among these, *PVT1* has been reported as a ceRNA that acts as an oncogenic molecule in stomach cancer^23^, involved in the occurrence and development of STAD through various biological processes such as EMT^24^, tumor angiogenesis^25^, and anti-5-fluorouracil resistance^26^. *KCNQ1OT1* has also been reported on the role of ceRNA^27^. Studies have found that *KCNQ1OT1* enhanced cell proliferation and migration in bladder cancer by modulating the *miR-145-5p/PCBP2* pathway^28^, and the interaction between *KCNQ1OT*1 and* miR-145-5p* was also found in this study. The latest experimental verification^29^ showed that *MAGI2-AS3* was a kind of lncRNA related to EMT, which maintains *ZEB1* overexpression by sponging *miR-141/200a*, leading to a poor prognosis of gastric cancer. However, our separate KM analysis did not demonstrate correlation between these three lncRNAs and ASTAD prognosis, perhaps because our study only targeted patients with advanced stages. Currently, researches on lncRNA by scholars mainly focus on bioinformatics analysis and basic experiments. Therefore, future investigations should encompass more clinical trials aimed at validating their clinical significance across different pathological stages.

In this study, we conducted KM survival analysis on 114 patients with stage III or IV STAD in the TCGA database. We found significant correlations between the lncRNAs AC125807.2, LINC02086, and LINC02535 from our ceRNA network and patient survival. Elevated expression levels of* LINC02086* and* LINC02535* predicted poor prognosis in ASTAD patients. So far, limited research has been conducted on these lncRNAs, with only one study linking *LINC02535* to gastric cancer development. The study by Jianzhong Wu et al.^30^ utilized transcriptome sequencing on clinical tissues from poorly differentiated gastric cancer patients found that* LINC02535* was differentially highly expressed in tumor tissues and verified its oncogenic function in vitro. Furthermore, another study suggested an association between *LINC02535* and cervical cancer^31^. Similarly, current studies have solely implicated LINC02086 as a ceRNA involved in hepatocellular carcinoma^32^ and laryngeal squamous cell carcinoma^33^. Therefore, to validate our bioinformatics findings, we performed RT-qPCR to determine the expression levels of *LINC02086* and *LINC02535*, which demonstrated a substantial up-regulation of their expression in gastric cancer cells, aligning with our bioinformatics prediction.

Further, in accordance with the ceRNA action model, a positive correlation is expected to exist between the expression levels of lncRNAs and their competing mRNAs within the network. In our constructed ceRNA network model, most lncRNA-miRNA-mRNA expressions adhere to this rule. Notably, *MAGI2-AS3* and *ZCCHC24* exhibit the strongest correlation among the groups of lncRNAs and mRNAs. However, there were also some groups has low correlation coefficients or even opposite expression trends. This could be attributed to other molecules in gastric cancer influencing the expression of these specific mRNAs alongside the role played by lncRNA as a ceRNA. Considering our aim to explore the specific possible mechanism of ceRNA in ASTAD, and based on the PCR results indicating high expression of that *LINC02086* and *LINC0253* in GC cells, we screened mRNAs in the ceRNA that were also highly expressed in ASTAD, and selected *MMP3* with the highest log_2_FC value as the key mRNA for further study. In the ceRNA network, *MMP3* and *LINC02086* could competitively bind *miR-93-5p*, as directly confirmed by our dual luciferase reporter assay in vitro, and in the *LINC02086* knock down gastric cancer cells, *MMP3* was accordingly down-regulated in both mRNA and protein levels. In addition, for *LINC02086*, our study also confirmed that its knock down has biological functions including cell cycle arrest, inhibiting cell proliferation and metastasis in gastric cancer cells. These results imply that the *LINC02086*/miR-93-5p*/MMP3* mechanism pathway may contribute to the pathogenesis of ASTAD. Functional enrichment analysis of DEGs in the ceRNA network revealed that it promotes cancer by interfering with gene transcription and cell proliferation, aligning with our in vitro experimental results. However, animal experiments are needed to confirm these findings, which will be conducted in our ongoing research.

Current studies suggest that ceRNA networks can be used for survival risk assessment in tumors. For instance, multiple lncRNA biomarkers have been identified to predict prognosis in colon cancer^34^, while 5-lncRNA has shown potential as a prognostic biomarker for survival prediction in breast cancer^35^. To assess the prognostic value of our ceRNA network in ASTAD, we examined the risk scores of 6 lncRNAs through COX regression and performed survival analysis in the TCGA-ASTAD training cohort and the GSE154595 external validation cohort. Our results showed that ceRNA network model can serve as a prognostic model for ASTAD, with lncRNAs being potential biomarkers. Specifically, to our knowledge, this is the first study to develop a lncRNA risk score for advanced stomach adenocarcinoma utilizing the ceRNA network. However, our study has limitations, as it relies on ceRNA theory and requires further experimental validation and clinical follow-up.

## Conclusions

Based on differentially expressed mRNAs, miRNAs, and lncRNAs in advanced stomach cancer, we created a ceRNA network in this study. Thus, we determined a 6-lncRNA network model* (PVT1, MAGI2-AS3, KCNQ1OT1, LINC02086, AC125807.2* and *LINC02535*) that could predict survival risk and prognosis of ASTAD. And through GO and KEGG analysis of differentially expressed mRNAs, we found evidence suggesting that ceRNA may bind to miRNAs with target genes to regulate gene expression and interfere with the gene transcription and proliferation of tumor cells. Notably, *LINC02086* and *LINC02535* showed potential as poor prognostic signatures for ASTAD patients. Finally, we determined that LINC02086 has biological effects that promote the proliferation and migration of gastric cancer cells. This finding may be connected to a putative method of regulation of *LINC02086*/miR-93-5p/MMP3 as a ceRNA. Overall, this study constructs a ceRNA network of ASTAD and provides valuable information for further understanding of its molecular mechanism.

## Supplementary Material

Supplementary figure and tables.

## Figures and Tables

**Table 1:** The mutual prediction results between LncRNA-miRNA and miRNA-mRNA from gdcCEAnalysis package in R and supplemented by Starbase database, miRTarBase and TargetScan databases online

LncRNA	miRNA	mRNA
PVT1	hsa-miR-93-5p	TNFRSF10B, SUSD1, MCM7, KAT2B, SLC2A4, E2F1, ZBTB4, CDKN1A, CXCL8, MMP3, FOXA1
PVT1	hsa-miR-106a-5p	TNFRSF10B, SUSD1
PVT1	hsa-miR-20a-5p	ILDR1, LAMP3, FHL1, DNMT1, RUNX1, TCEAL1, LIMK1, UBE2C, E2F1, KIT, RBL1, BCL2, CDKN1A, KIF26B, EGLN3, RGS5, BAMBI, PTPRO
PVT1	hsa-miR-106b-5p	LAMP3
MAGI2-AS3	hsa-miR-374b-5p	ID4, PPP1R3C, VAMP2, TLE4, LPAR1, NTN1, NOVA1, CYBRD1, ADAMTSL3, DUSP19, RECK, DMD, MEIS1, NR3C1, NFIX, TTLL7, MYLK, SATB1, FGF13, AKAP2, TNS1, PDZD2, UST, ZCCHC24, LRCH2, ATP8B2, L1CAM, MKX, PCDH7
MAGI2-AS3	hsa-miR-374a-5p	ID4, PPP1R3C, VAMP2, TLE4, LPAR1, NTN1, NOVA1, CYBRD1, ADAMTSL3, DUSP19, RECK, DMD, MEIS1, NR3C1, NFIX, TTLL7, MYLK, SATB1, FGF13, AKAP2, TNS1, PDZD2, UST, ZCCHC24, LRCH2, ATP8B2, L1CAM, MKX, PCDH7
KCNQ1OT1	hsa-miR-7-5p	HELLS
KCNQ1OT1	hsa-miR-140-5p	HELLS
KCNQ1OT1	hsa-miR-24-3p	HELLS, EML4
KCNQ1OT1	hsa-miR-29c-3p	MYBL2, EML4, COL5A2, C1QTNF6, SLC12A8, BMP1, MEST, LOXL2, PDGFRB, F11R, ADAMTS7, COL7A1, TMPRSS3, ITGA11, DIO2
KCNQ1OT1	hsa-miR-29b-3p	MYBL2, EML4, COL5A2, C1QTNF6, SLC12A8, BMP1, MEST, LOXL2, PDGFRB, F11R, ADAMTS7, COL7A1, TMPRSS3, ITGA11, DIO2
KCNQ1OT1	hsa-miR-29a-3p	MYBL2, EML4, COL5A2, C1QTNF6, SLC12A8, BMP1, MEST, LOXL2, PDGFRB, F11R, ADAMTS7, COL7A1, TMPRSS3, ITGA11, DIO2
KCNQ1OT1	hsa-miR-338-3p	EML4
KCNQ1OT1	hsa-miR-145-5p	EML4
KCNQ1OT1	hsa-miR-183-5p	EML4
KCNQ1OT1	hsa-miR-148b-3p	VAV2, ITGA11
KCNQ1OT1	hsa-miR-152-3p	VAV2, ITGA11
KCNQ1OT1	hsa-miR-148a-3p	VAV2, ITGA11
KCNQ1OT1	hsa-miR-335-5p	VAV2
LINC02086	hsa-miR-143-3p	LIMK1, COL3A1, HNF4A, GABARAPL1, BCL2, BAG3, MACC1, MMP9, SERPINE1, HK2, TLR2, KLF5
LINC02086	hsa-miR-93-5p	TNFRSF10B, SUSD1, MCM7, KAT2B, SLC2A4, E2F1, ZBTB4, CDKN1A, CXCL8, MMP3, FOXA1
LINC02086	hsa-miR-17-5p	SLC16A9, KAT2B, DNMT1, RUNX1, BRCA2, TCEAL1, UBE2C, E2F1, TNFSF12, RBL1, ZBTB4, BCL2, CDKN1A, VLDLR, IGFBP3, NPAS3, CLU, TIMP3, PTPRO
LINC02086	hsa-miR-20a-5p	ILDR1, LAMP3, FHL1, DNMT1, RUNX1, TCEAL1, LIMK1, UBE2C, E2F1, KIT, RBL1, BCL2, CDKN1A, KIF26B, EGLN3, RGS5, BAMBI, PTPRO
LINC02086	hsa-miR-20b-5p	NCAPG, CCNB1, NCAPG2, BRCA1, CDKN1A, BAMBI
AC125807.2	hsa-let-7c-5p	TNFRSF10B, CDC25A, HMGA2, IL6
LINC02535	hsa-miR-30a-5p	NCAM1, DNMT1, SOX4, DTL, KLF9, CCNE2, HNF4G, SKP2, LOX, BCL11A, CDH1, EYA2, IL21R

## Abbreviations

ASTAD: advanced stomach adenocarcinoma
GC: gastric cancer
lncRNA: long noncoding RNA
ceRNA: competing endogenous RNA
TCGA: The Cancer Genome Atlas
OS: overall survival
DEG: differentially expressed
DELnc: differentially expressed lncRNA
DEM: differentially expressed miRNA
GO: gene ontology
KEGG: Kyoto Encyclopedia of Genes and Genomes
BP: biological processes
CC: cellular composition
MF: molecular function
KM: Kaplan Meier
AUC: area under the curve
EMT: Epithelial-Mesenchymal Transition
