# Supplementary material for: Integrated Bioinformatics and Experimental Analysis of Long Noncoding RNA Associated-ceRNA as Prognostic Biomarkers in Advanced Stomach Adenocarcinoma
Source: J Cancer. 2024 Jan 21;15(6):1536–50. doi: 10.7150/jca.89526 (PMC10869988; doi:10.7150/jca.89526)
Supplement: Supplementary file 1 — Supplementary figure and tables. [file jcav15p1536s1.zip › supplementary meterial/Supplemental Tables 1-2.docx]

**Supplemental Table 1. Clinical characteristics of 186 advanced stomach adenocarcinoma patients**

| patients | N | 186 |
| --- | --- | --- |
| Stage *N* (%) | III | 148 (79.57%) |
|  | IV | 38 (20.43%) |
| Age *N* (%) | ≤ 70 | 125 (67.20%) |
|  | ＞70 | 61(32.80%) |
| Gender *N* (%) | Male | 116 (62.37%) |
|  | Female | 70 (37.63%) |
| Race *N* (%) | Asian | 30 (16.13%) |
|  | Black or African American | 7 (3.76%) |
|  | White | 118 (63.44%) |
|  | Native hawaiian or other pacific islander | 1 (0.54%) |
|  | Not reported | 30 (16.13%) |
| Duration *N* (%) | ＜5 years | 180 (96.77%) |
|  | ≥ 5years | 6 (3.23%) |

**Supplemental Table 2 The top 10 protein coding mRNAs with the most significantly different expression were found in patients with advanced gastric adenocarcinoma.**

| ID | symbol | group | log_2_FC | AveExpr | t | PValue | FDR | B |
| --- | --- | --- | --- | --- | --- | --- | --- | --- |
| ENSG00000149968 | MMP3 | protein_coding | 3.761801 | 2.104338 | 4.240804 | 3.97E-05 | 0.000176 | 1.867487 |
| ENSG00000101076 | HNF4A | protein_coding | 3.443495 | 6.112577 | 6.519076 | 1.12E-09 | 1.73E-08 | 11.61996 |
| ENSG00000175063 | UBE2C | protein_coding | 3.029968 | 5.131396 | 7.352879 | 1.36E-11 | 3.58E-10 | 15.96675 |
| ENSG00000149948 | HMGA2 | protein_coding | 2.939904 | 2.174781 | 5.01525 | 1.54E-06 | 9.88E-06 | 4.899385 |
| ENSG00000169429 | CXCL8 | protein_coding | 2.790805 | 4.596791 | 4.296571 | 3.18E-05 | 0.000145 | 1.880608 |
| ENSG00000164749 | HNF4G | protein_coding | 2.629091 | 4.528646 | 5.580602 | 1.16E-07 | 1.01E-06 | 7.219197 |
| ENSG00000100985 | MMP9 | protein_coding | 2.586051 | 4.206423 | 5.294415 | 4.39E-07 | 3.28E-06 | 5.972159 |
| ENSG00000145103 | ILDR1 | protein_coding | 2.564461 | 2.524829 | 6.534226 | 1.04E-09 | 1.62E-08 | 11.8404 |
| ENSG00000109805 | NCAPG | protein_coding | 2.478039 | 3.723377 | 7.755973 | 1.49E-12 | 5.17E-11 | 18.1466 |
| ENSG00000143476 | DTL | protein_coding | 2.350529 | 3.798182 | 8.865309 | 2.72E-15 | 2.34E-13 | 24.26693 |
