# Supplementary figures and images for: Integrated Bioinformatics and Experimental Analysis of Long Noncoding RNA Associated-ceRNA as Prognostic Biomarkers in Advanced Stomach Adenocarcinoma
Source: J Cancer. 2024 Jan 21;15(6):1536–50. doi: 10.7150/jca.89526 (PMC10869988; doi:10.7150/jca.89526)

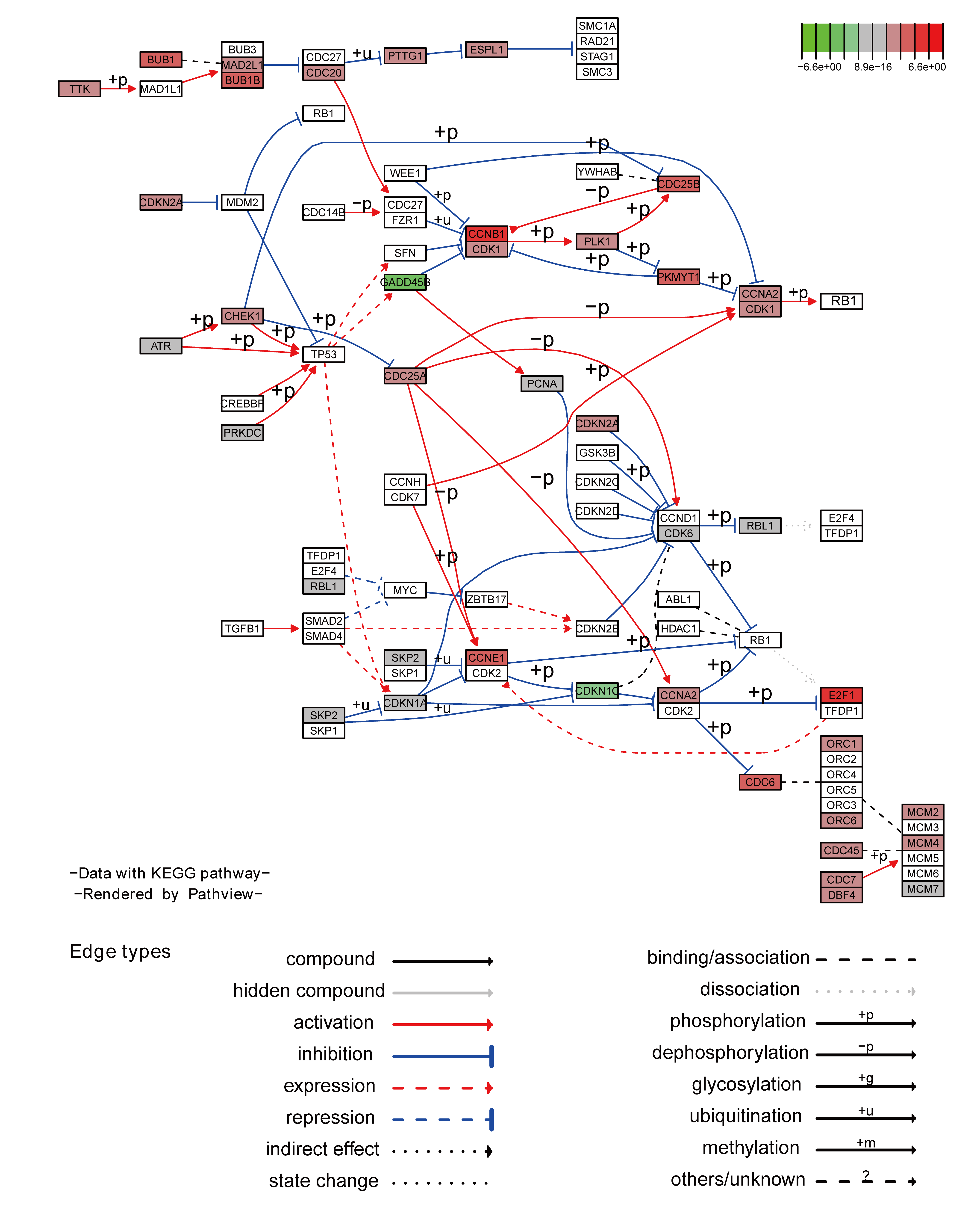

Supplement: Supplementary file 1 — Supplementary figure and tables. [file jcav15p1536s1.zip › supplementary meterial/Supplemental Figure 1.tif]
